# Frequency and factors associated with hospital readmission after COVID-19 hospitalization: the importance of post-COVID diarrhea

**DOI:** 10.1016/j.clinsp.2022.100061

**Published:** 2022-06-13

**Authors:** Maristela Pinheiro Freire, Maura Salaroli Oliveira, Marcello Mihailenko Chaves Magri, Bruno Melo Tavares, Igor Marinho, Ana Catharina De Seixas Santos Nastri, Geraldo Filho Busatto, HCFMUSP COVID-19 Study Group, Anna S. Levin

**Affiliations:** a Working Committee for Hospital Epidemiology and Infection Control, Hospital das Clínicas, Faculdade de Medicina, Universidade de São Paulo (HCFMUSP), São Paulo, SP, Brazil; b Department of Infectious Diseases, Hospital das Clínicas, Faculdade de Medicina, Universidade de São Paulo (HCFMUSP), São Paulo, SP, Brazil; c Laboratory of Psychiatric Neuroimaging (LIM-21), Department and Institute of Psychiatry, Faculdade de Medicina, Universidade de São Paulo (HCFMUSP), São Paulo, SP, Brazil

**Keywords:** Long COVID, Diarrhea, Dysbiosis, Infection, Myalgia

## Abstract

**Purpose:**

The aim of this study was to describe the incidence and risk factors for hospital readmission and infection during the months after COVID-19 hospital admission.

**Methods:**

This prospective study included adult patients who were hospitalized due to COVID-19 and had been discharged from April 2020 to August 2020. All patients had a medical evaluation with a structured questionnaire 6 to 11 months after hospital admission. The authors included only patients with confirmed COVID-19 by RT-PCR. Patients with pregnant/postpartum women, with a proven COVID-19 reinfection or incapable of answering the questionnaire were excluded.

**Results:**

A total of 822 patients completed the follow-up assessment, and 68% reported at least one recurrent symptom related to COVID-19. The most frequent symptom was myalgia (42%). Thirty-two percent of patients visited an emergency room after COVID-19 hospitalization, and 80 (10%) patients required re-hospitalization. Risk factors for hospital readmission were orotracheal intubation during COVID-19 hospitalization (p = 0.003, OR = 2.14), Charlson score (p = 0.002, OR = 1.21), congestive heart failure (p = 0.005, OR = 2.34), peripheral artery disease (p = 0.06, OR = 2.06) and persistent diarrhea after COVID-19 hospitalization discharge (p = 0.02, OR = 1.91). The main cause of hospital readmission was an infection, 43 (54%). Pneumonia was the most frequent infection (29%).

**Conclusions:**

The presence of symptoms after six months of COVID-19 diagnosis was frequent, and hospital readmission was relatively high.

## Highlights

• 32% of the patients visited an emergency room after COVID-19 hospitalization.
• The rate of hospital readmission after COVID-19 hospitalization is high, in the present sample 10% of patients needed a second hospitalization in 6-months
• Patients with persistent diarrhea after COVID-19 discharge had two times more chance to have another hospitalization in the next 6-months.

## Background

The pandemic of Coronavirus Disease 2019 (COVID-19) caused by SARS-CoV-2 continues to affect all levels of the health care system. Globally, there have been 226,844,344 cases of this highly contagious respiratory disease; and 4,666,334 deaths. In Brazil, from 3 January 2020 through 17 September 2021, there were 21,034,610 confirmed cases with 588,597 deaths, as reported to the World Health Organization.^1^

COVID-19 is a disease that affects multiple organs and systems with a broad spectrum of acute, subacute, and long-term manifestations. Symptoms of acute COVID-19 infection include cough, fever, fatigue, diarrhea, pneumonia, and dyspnea.^2^

The majority of people who acquire COVID-19 have a mild or moderate disease. However, severe respiratory symptoms may lead to life-threatening respiratory failure, resulting in the urgent need for invasive ventilation in an intensive care unit. More severe diseases occur particularly in people over 65-years of age and with comorbidities such as cardiovascular and chronic respiratory diseases, as well as diabetes.^3^

Beyond the acute disease, several studies reported persistent symptoms. Patients recovering from severe COVID-19 are likely to have undergone a prolonged period of immobilization, sedation, and ventilation. Patients who have experienced prolonged hospitalization in intensive care units may present sarcopenia, muscle weakness, general deconditioning, myopathy, and neuropathy, as well as musculoskeletal dysfunction.^4^, ^5^, ^6^ Also, COVID-19 infection and inflammation are related to increasing cases of Post-COVID Neurological Syndrome (PCNS).^7^

In a study using a questionnaire to evaluate patients who had had COVID-19, most of the subjects who recovered from COVID-19 presented frequent mild symptoms such as fatigue, anxiety, joint pain, and headache, or more critical manifestations such as pulmonary fibrosis, stroke, and myocarditis. The severity of post-COVID-19 manifestations was correlated to the severity of the infection which also was related to the presence of comorbidities.^8^

Therefore, post-COVID-19 symptoms are common and may affect the quality of life of patients well as the procurement of healthcare assistance. Determining the main risk population for developing the post-COVID-19 symptoms could contribute to designing measures for prevention, and prepare the healthcare system for this additional demand. Thus, the goal of this study was to identify factors associated with hospital readmission, and specifically hospital readmission due to infection during the period after COVID-19 hospital discharge.

## Methods

### Study design and patients

All patients aged ≥18 years who had been admitted (for at least 24 hours) as inpatients to Hospital das Clínicas da Faculdade de Medicina da Universidade de São Paulo (HC-FM-USP), Brazil, due to laboratory-confirmed COVID-19 between March and August 2020 were consecutively invited for a follow-up in-person visit between October 2020 and April 2021. Exclusion criteria were pregnant/postpartum women, dementia, end-stage cancer, subjects living in nursing homes or long-term care facilities, patients with insufficient physical mobility to leave home and suspected current COVID-19 re-infection. Hospital das Clinicas (HC) is a 2,400-bed university hospital that during the COVID-19 pandemic had a 600-bed building dedicated to COVID-19 care.

All patients received a telephone call after discharge inviting them to return for an outpatient evaluation approximately 6 to 11 months after hospitalization. During this consultation, a trained physician applied a structured questionnaire created by a multidisciplinary team. If a patient was unable to come to the hospital, he was invited to participate through telehealth.^9^ The authors defined persistent COVID-19 symptoms such as diarrhea, cough, or body pain. All these symptoms were only recorded if the patient did not present that symptom before COVID-19. When the patient described an infectious episode, the interviewer detailed as well as possible its clinical presentation, microbiological workup, and treatment based on the patient's information. An episode of fever without any other symptom was recorded as a new episode of infection.

Data from interviews, evaluations and complementary examinations were captured and stored in real-time using a web-based case report form developed on a Research Electronic Data Capture (REDCap) system hosted at HC. Hospitalization data were obtained from electronic patients’ records. The authors considered the end of follow-up as the date of the outpatient evaluation.

The information regarding hospital readmission and infection was collected during the interviews. All patients who reported re-hospitalization or infection had their electronic records and microbiologic tests reviewed by the investigators.

### Definitions

Diarrhea was defined as the passage of three or more loose or liquid stools per day (or more frequent passage than is normal for the individual).^10^

The authors classified educational level into 4 levels: 1 – Illiterate; 2 – Incomplete primary school, 3 – Complete middle school; 4 – Any level of high school.

A sedentary lifestyle was defined as that of patients classified in category 1 of the International Physical Activity Questionnaire.^11^

The social-economic classification used was that outlined by the Brazilian Market Research Association.^12^

### Missing data

Missing data for independent variables varied from zero to 4.3%. Data were assumed to be missing at random conditional on the observed variables. A monotone missing data pattern and regression were used to generate multiple imputed datasets according to the different outcomes.

### Data analysis

The authors analyzed two main outcomes, hospital readmission, and hospital readmission due to infection. The authors defined hospital readmission due to infection as the patient had the diagnosis of infection on admission. In the analysis of risk factors for hospital readmission due to infection, patients who had been readmitted due to other causes were excluded. Persistent symptoms (cough, myalgia, and diarrhea) were recorded until hospital readmission or until the follow-up evaluation for patients without this outcome.

In the statistical analysis, we used the Chi-Square test or Fisher's exact test, as indicated, for dichotomous variables, whereas the Mann-Whitney test was used for continuous variables. Variables showing a value of p < 0.2 in univariate analysis were included in a multivariate analysis performed by stepwise logistic regression. Continuous variables were transformed into dichotomous variables through cluster analysis and the ones with the lowest p-value were included in the analysis. Multicollinearity was tested using the variance inflation factor. Variables that then reduced the −2 log-likelihood or showed a value of p < 0.05 were retained in the model.

## Results

**Figure 1:**
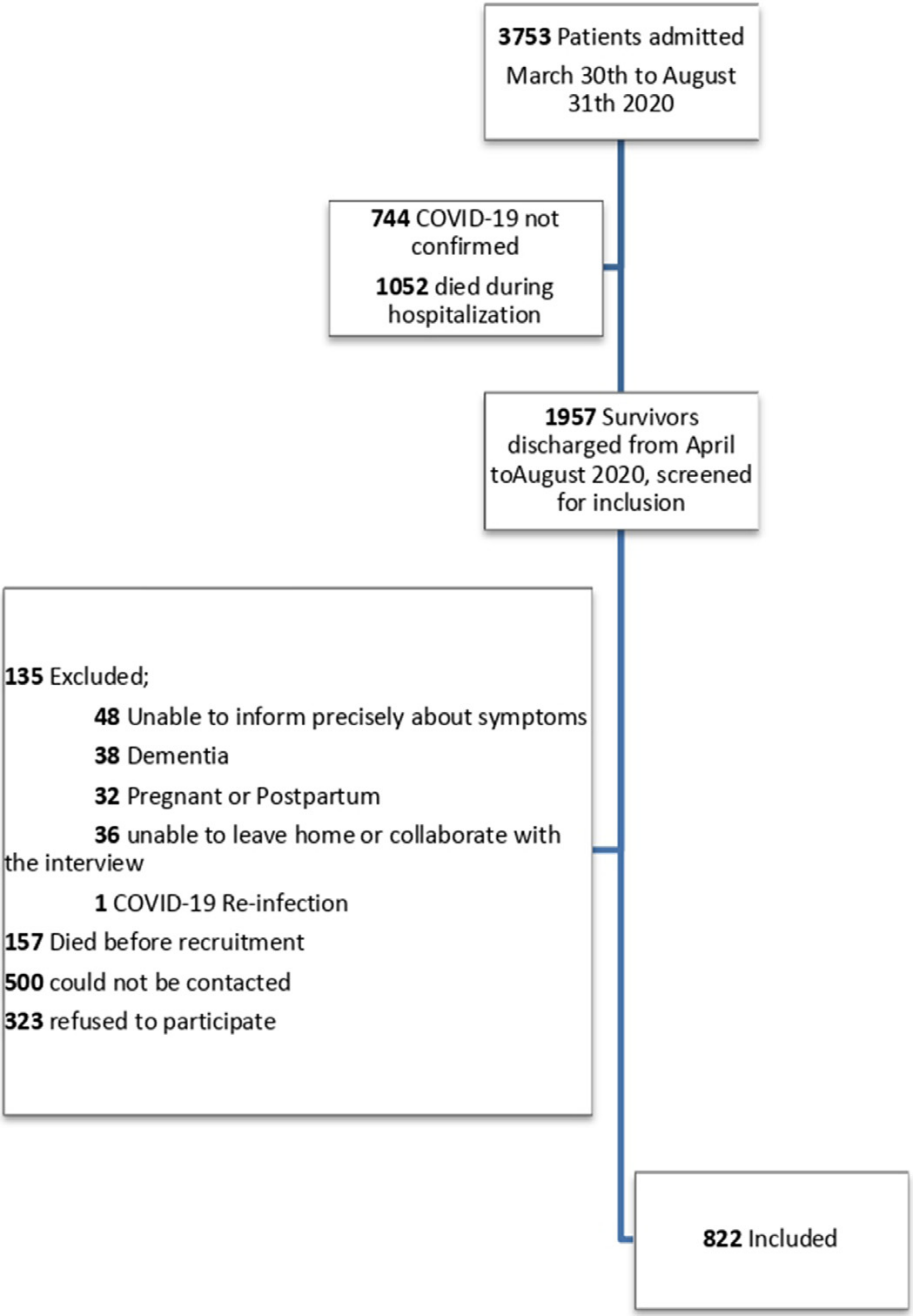
Flowchart of potentially eligible subjects and final sample included in the follow-up of patients discharged from hospitalization due to COVID-19.

1957 patients with COVID-19 survived hospitalization and 822 of these completed the follow-up assessment (Fig. 1). Fifty- three percent were male with a median age of 56 years (range: 18 to 101 years). The most common comorbidity was systemic arterial hypertension (57%); 63% required admission to ICU, and 41% underwent mechanical ventilation (Table 1). A linear relationship was observed between the Charlson score and age (p < 0.001) (Supplementary Fig. 1).

**Table 1:** Characteristics of 822 patients evaluated 6 to 11-months after admission from COVID-19 hospitalization.

Variables	n	
**On hospital admission for COVID-19**		
Age in years (median, range)	56	18‒101
Male sex	437	53%
Systemic Arterial Hypertension	471	57%
Diabetes mellitus	296	36%
Chronic kidney disease	106	13%
Congestive heart failure	103	13%
Chronic liver disease	47	6%
Chronic obstructive pulmonary disease	44	5%
Cancer	41	5%
Asthma	30	4%
**COVID-19 hospitalization**		
Days of hospital stay (median, range)	13	1‒163
Admission to ICU	516	63%
Orotracheal intubation	339	41%
Dialysis during hospital stay	104	13%
**Persistent symptoms after COVID-19 hospital discharge**		
Myalgia	342	42%
Cough	324	39%
Diarrhea	184	22%
Any of the three symptoms	558	68%
All 3 symptoms	60	7%
Need for domiciliary oxygen	21	3%
**Outcomes after discharge from COVID-19 hospitalization**		
Self-reported Infection	177	22%
Al least one visit to Emergency Room	223	27%
Hospital readmission	80	10%
Hospital readmission due to Infection	43	5%

The median time from hospital discharge to the follow-up evaluation was 200 days (range: 90‒380 days). During the follow-up consultation, patients presented with median oxygen saturation of 97% (range: 80%‒100%) and mean arterial pressure of 93 mmHg (range: 69–140 mmHg).

A high proportion of patients (68%) reported at least one persistent symptom related to COVID-19 (myalgia, cough, or body pain). The most frequent symptom was myalgia 42%, followed by cough 39% (Table 1).

The three symptoms were associated, i.e., patients who had diarrhea had a relative risk of presenting a cough of 1.50 (p < 0.001) and 1.80 of having myalgia (p < 0.001).

The median duration of myalgia was 191 days (range: 1‒323), median cough duration was 61 days (range: 1‒344), and diarrhea was 16 days (range: 1‒302).

Infections after discharge were reported in 177 patients (22%), the most common infection reported was flu-like syndrome (34%), followed by urinary tract infections (28%); and pulmonary infection (13%). Three patients had more than one infection during the follow-up period. (Supplementary Fig. 2)

**Figure 2:**
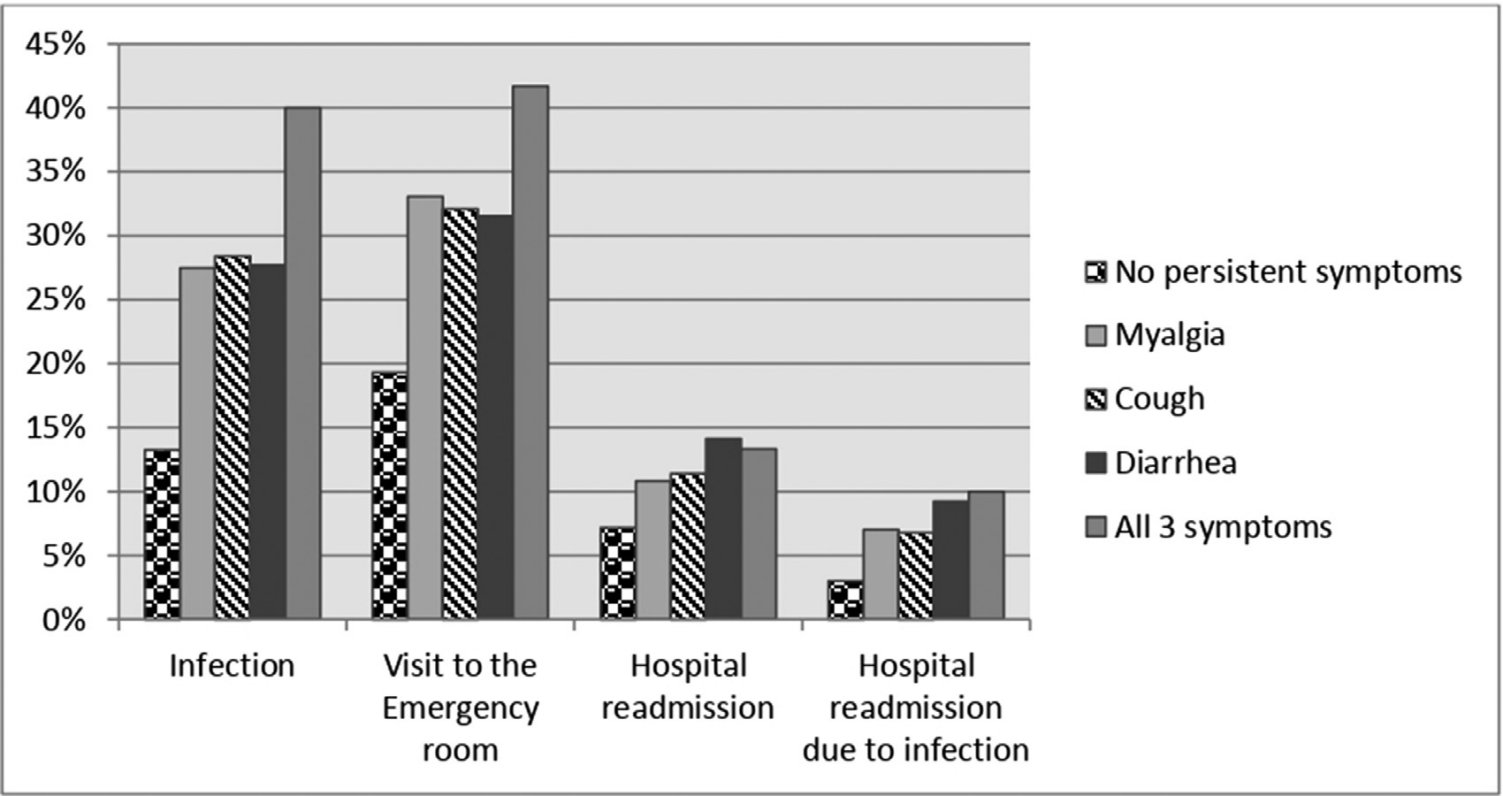
Frequency of symptoms among patients who required medical assistance after discharge from COVID-19 hospitalization.

Approximately one-third (27%) reported visits to an Emergency Room (ER). Patients who reported persistence of COVID-19 symptoms had a higher risk of going to ER after hospital discharge (p = 0.001). The persistence of COVID-19 symptoms increased the risk of self-reported infection, visits to the ER, and hospital readmission (Fig. 2).

Hospital readmission occurred in 80 (10%) patients. Independent risk factors for hospital readmission were orotracheal intubation during COVID-19 hospitalization (p = 0.003, OR = 2.14), Charlson score (p = 0.002, OR = 1.21), congestive heart failure (p = 0.005, OR = 2.34), peripheral artery disease (p = 0.06, OR = 2.06) and persistent diarrhea after COVID-19 hospitalization discharge (p = 0.02, OR = 1.91) (Table 2).

**Table 2:** Risk factors for hospital readmission after discharge from hospitalization due to COVID-19.

					Multivariate analysis	
	Hospital readmission (n = 80)	Were not readmitted (n = 742)	RR (95% CI)	p-value	OR (95% CI)	p-value
Age in years median (range)	53 (20‒91)	57 (18‒101)	‒	0.62		
Male gender	39 (49%)	398 (54%)	0.98 (0.94‒1.03)	0.41		
**Educational level**						
1	34 (43%)	256 (35%)	‒	0.37		
2	15 (19%)	133 (18%)	‒			
3	19 (24%)	238 (32%)	‒			
4	6 (8%)	98 (13%)	‒			
BMI on hospital admission	31.2 (16.6‒63.3)	30.7 (13.6‒67.3)	‒	0.83		
**Variables associated with COVID-19 hospitalization**						
WHO severity class	4 (1‒4)	2 (1‒4)	‒	0.11		
Days of hospital stay median (range)	18 (1‒154)	13 (1‒163)	‒	0.01		
Admission to ICU	50 (63%)	440 (59%)	1.05 (1.01‒1.09)	0.06		
Days of ICU stay Median (range)	7 (0‒76)	4 (0‒126)	‒	0.04		
Orotracheal intubation	43 (54%)	296 (40%)	1.06 (1.01‒1.11)	0.02	2.14 (1.31‒3.51)	0.003
Dialysis	11 (14%)	93 (13%)	1.01 (0.94‒1.08)	0.76		
**Underlying diseases**						
Charlson score	4 (1‒10)	2 (1‒10)	‒	0.002	1.21 (1.08‒1.37)	0.002
Systemic Arterial Hypertension	51 (64%)	420 (57%)	1.03 (0.98‒1.08)	0.22		
Chronic obstructive pulmonary disease	8 (10%)	36 (5%)	1.11 (0.96‒1.28)	0.05		
Asthma	3 (4%)	27 (4%)	1.00 (0.89‒1.13)	>0.99		
Chronic kidney disease	16 (20%)	90 (12%)	1.07 (0.99‒1.17)	0.05		
Smoking	28 (35%)	285 (38%)	0.99 (0.94‒1.03)	0.55		
Rheumatic disease	20 (25%)	207 (28%)	0.99 (0.94‒1.04)	0.58		
Hematologic disease	0	16 (2%)	0.89 (0.87‒0.92)	0.40		
Diabetes mellitus	38 (48%)	258 (35%)	1.06 (1.00‒1.11)	0.02		
Cancer	7 (9%)	34 (5%)	1.09 (0.95‒1.26)	0.10		
Sleep apnea	9 (11%)	128 (17%)	0.96 (0.91‒1.01)	0.17		
Coronary heart disease	18 (23%)	183 (25%)	1.11 (1.01‒1.22)	0.003		
Congestive heart failure	20 (25%)	83 (11%)	1.14 (1.03‒1.25)	<0.001	2.34 (1.29‒4.23)	0.005
Peripheral artery disease	10 (13%)	53 (7%)	1.08 (0.97‒1.20)	0.09	2.06 (0.97‒4.38)	0.06
Acute arterial occlusion	1 (1%)	8 (1%)	1.02 (0.81‒1.28)	0.60		
Thromboembolic events during hospital stay	3 (4%)	49 (7%)	0.96 (0.89‒1.03)	0.47		
Chronic liver disease	7 (9%)	40 (5%)	1.06 (0.94‒1.20)	0.22		
Sedentary lifestyle	40 (50%)	279 (38%)	1.06 (1.01‒1.11)	0.01		
**Social economic class**						
A	1 (1%)	19 (3%)	‒	0.18		
B1	4 (5%)	39 (5%)	‒			
B2	9 (11%)	135 (18%)	‒			
C1	28 (35%)	239 (32%)	‒			
C2	29 (36%)	212 (29%)	‒			
D	3 (4%)	78 (11%)	‒			
**Symptoms after hospital discharge**						
Diarrhea	26 (33%)	158 (21%)	1.07 (1.01‒1.14)	0.02	1.91 (1.13‒3.23)	0.02
Myalgia	37 (46%)	305 (41%)	1.02 (0.98‒1.07)	0.38		
Cough	37 (46%)	287 (39%)	1.03 (0.98‒1.08)	0.19		
Chest pain	27 (34%)	186 (25%)	1.05 (0.99‒1.11)	0.08		
All 3 symptoms (Diarrhea, myalgia, cough)	8 (10%)	52 (7%)	1.05 (0.94‒1.16)	0.33		
Any of the 3 symptoms (Diarrhea, myalgia, cough)	61 (76%)	497 (67%)	1.04 (1.00‒1.09)	0.09		

RR, Relative Risk; OR, Odds Ratio; CI, Confidence Interval; BMI, Body Mass Index; ICU, Intensive Care Unit.

The main cause of hospital readmission was an infection, 43 (54%). The most common site of infection among patients who need hospitalization was pneumonia (30%), urinary tract infection (26%), and flu-like syndrome (16%). Among patients who had flu-like symptoms or fever, 63% were tested for SARS-Cov-2 (by RT-PCR), and all were negative. Independent risk factors for hospital readmission due to infection were orotracheal intubation during COVID-19 hospitalization (p = 0.05), high BMI at COVID-19 hospital admission (p = 0.05), high Charlson score at COVID-19 hospital admission (p = 0.006), peripheral artery disease (p = 0.02), and diarrhea during the follow-up (p = 0.005) (Table 3). When patients with hospital readmission due to infection were compared to patients with readmission for other causes the only significant difference was that patients with infection had more diarrheal episodes after their COVID-19 discharge (47% vs. 22%, respectively; p = 0.02)

**Table 3:** Risk factors for hospital readmission due to infection after COVID-19 hospitalization.

					Multivariate analysis	
	Readmission due to infection (n = 43)	Not readmitted to the hospital (n = 778)	RR (95% CI)	p-value	OR (95% CI)	p-value
Age in years median (range)	61 (20‒83)	56 (18‒101)	‒	0.48		
Male gender	21 (49%)	415 (53%)	0.99(0.96‒1.02)	0.56		
Educational level				0.66		
1	17 (40%)	273 (35%)	‒			
2	9 (21%)	139 (18%)	‒			
3	13 (30%)	244 (31%)	‒			
4	3 (7%)	101 (13%)	‒			
BMI at hospital admission	34 (19‒63)	31 (14‒67)	‒	0.04	1.04 (1.00‒1.09)	0.05
**Variables associated with COVID-19 hospitalization**						
WHO severity class	4 (1‒4)	2 (1‒4)	‒	0.02		
Days of hospital stay median (range)	18 (1‒154)	13 (1‒163)	‒	0.09		
Admission to the ICU	30 (70%)	485 (62%)	1.02 (0.99‒1.05)	0.33		
Days of ICU stay median (range)	7 (0‒76)	3 (0‒126)	‒	0.04		
Orotracheal intubation	25 (58%)	313 (40%)	1.04 (1.00‒1.08)	0.02	2.22 (1.14‒4.33)	0.05
Dialysis	4 (9%)	100 (13%)	0.98 (0.94‒1.03)	0.64		
**Underlying diseases**						
Systemic arterial hypertension	29 (67%)	441 (57%)	1.02 (0.99‒1.06)	0.17		
Charlson score at hospital admission	4 (1‒9)	3 (1‒10)	‒	0.02	1.26 (1.07‒1.48)	0.006
Chronic obstructive pulmonary disease	6 (14%)	38 (5%)	1.10 (0.98‒1.24)	0.01		
Asthma	1 (2%)	29 (4%)	0.98 (0.92‒1.05)	0.63		
Chronic kidney disease	5 (12%)	101 (13%)	1.00 (0.95‒1.04)	0.80		
Smoking	19 (44%)	294 (38%)	1.01 (0.98‒1.05)	0.40		
Rheumatic disease	12 (28%)	215 (28%)	1.00 (0.97‒1.04)	0.97		
Sleep apnea	8 (19%)	129 (17%)	1.01 (0.96‒1.05)	0.73		
Hematologic disease	0	16 (2%)	0.94 (0.92‒0.96)	>0.99		
Diabetes mellitus	18 (42%)	278 (36%)	1.01 (0.98‒1.05)	0.42		
Cancer	4 (9%)	37 (5%)	1.05 (0.95‒1.17)	0.27		
Coronary heart disease	8 (19%)	93 (12%)	1.03 (0.97‒1.10)	0.20		
Congestive heart failure	11 (26%)	92 (12%)	1.07 (1.00‒1.15)	0.008	2.09 (0.96‒4.55)	0.06
Peripheral artery disease	7 (16%)	55 (7%)	1.07 (0.98‒1.18)	0.03	2.86 (1.16‒7.05)	0.02
Acute arterial occlusion	0	9 (1%)	0.95 (0.93‒0.96)	>0.99		
Thromboembolic events during hospital stay	2 (5%)	50 (%)	0.99 (0.93‒1.04)	>0.99		
Chronic liver disease	4 (9%)	43 (6%)	1.04 (0.95‒1.13)	0.30		
Sedentary lifestyle after COVID-19	25 (58%)	295 (6%)	1.05 (1.01‒1.08)	0.008		
**Social economic class**				0.28		
A	1 (2%)	19 (2%)	‒			
B1	3 (7%)	40 (5%)	‒			
B2	3 (7%)	141 (18%)	‒			
C1	16 (37%)	251 (32%)	‒			
C2	17 (40%)	224 (29%)	‒			
D	2 (5%)	79 (10%)	‒			
**Symptoms after hospital discharge**						
Diarrhea	17 (40%)	167 (21%)	1.06 (1.01‒1.11)	0.006	2.60 (1.34‒5.06)	0.005
Myalgia	24 (56%)	317 (41%)	1.03 (1.00‒1.07)	0.05		
Chest pain	16 (37%)	197 (25%)	1.03 (0.99‒1.08)	0.08		
Cough	22 (51%)	302 (39%)	1.03 (0.99‒1.06)	0.11		
All 3 symptoms (diarrhea, myalgia, cough)	6 (14%)	54 (7%)	1.06 (0.97‒1.15)	0.09		
Any of the 3 symptoms (diarrhea, myalgia, cough)	35 (81%)	522 (67%)	1.04 (1.00‒1.07)	0.51		

RR, Relative Risk; OR, Odds Ratio; CI, Confidence Interval; BMI, Body Mass Index; ICU, Intensive Care Unit.

Diabetes mellitus, female gender, and younger age were significantly associated with the presence of diarrhea after COVID-19 discharge. (Supplementary Table 1)

## Discussion

This prospective longitudinal study with more than 800 patients showed that a significant proportion of patients maintained a wide range of symptoms after discharge from COVID-19 hospitalization, requiring visits to the emergency department, and even hospitalization. The most frequent symptoms were myalgia, followed by cough, and diarrhea.

Persistence of symptoms, such as in the present study has already been observed. A recently published systematic review and meta-analysis estimated that 80% of COVID-19 patients developed one or more long-term symptoms, and the five most frequent symptoms were fatigue, headache, attention disorder, hair loss, and dyspnea.^13^

The present findings show that in at least one-third of the patients with persistent symptoms, seek medical assistance at the Emergency Department (ER) or required a new hospitalization; probably these post-COVID symptoms were part of the cause for this. In Brazil, as of August 2021, there have been 21 million confirmed cases of COVID-19. Thus, the need for ER attendance and hospital readmission will be an extra burden on the healthcare system, which is already operating at full capacity due to the care of COVID-19 acute cases, and due to the increased demand for patients with other diseases and whose healthcare was delayed due to the pandemic.^1^

Risk factors for developing post-COVID-19 symptoms are not well defined. The present study's data demonstrated that the presence of previous comorbidities such as a high Charlson score at COVID-19 hospital admission, heart failure, and peripheral artery disease were risk factors for readmission to the hospital. This is consistent with data that showed that a prior diagnosis of heart failure increased four times the risk of readmission within 60 days after discharge.^14^ Increased Charlson score has been associated with a higher risk of readmission in other diseases, and in COVID-19 the most common comorbidity associated with readmission is diabetes mellitus.^15^^,^^16^

Another point is that the need for mechanical ventilation during COVID-19 was significantly associated with the risk of readmission. A considerable proportion of patients who had severe COVID-19 showed radiological evidence of pulmonary abnormalities and pulmonary function tests exhibiting restrictive functional deficits that persist months after recovery from acute COVID-19 disease.^17^ A German cohort described that 32% of patients who required mechanical ventilation during COVID-19 hospitalization were readmitted to a hospital in 180-days.^18^ Patients who required mechanical ventilation certainly are at a greater risk of having pulmonary sequelae and the authors hypothesized that these may be responsible for hospital readmission. In other words, it seems there is a relationship between the risk of readmission and the severity of the disease, indicated by the need for mechanical ventilation.^13^

Surprisingly, the presence of diarrhea in the follow-up evaluation was significantly associated with the risk of hospital readmission. Diarrhea has been described as a common persistent symptom. Some authors consider that COVID-19 alters the gut microbiome, and these microbiome alterations have been associated with the severity of COVID-19. In addition, specific groups of bacterial species that can downregulate the expression of ACE2 in murine gut studies were found in abundance in fecal samples with high SARS-CoV-2 load.^19^, ^20^, ^21^ Additionally, patients with COVID-19 who require hospitalization are submitted to several factors that promote dysbioses such as antibiotic use, proton pump inhibitor use, dysglycemia, and obesity.^22^ Therefore, the persistence of diarrhea may be associated with the inability of the patients to recover a healthy intestinal microbiome. Finally, dysbiosis is a well-known risk for infections, cardiovascular events, and inflammatory illness, all causes of hospital readmission.^22^ Some studies suggest that the persistence of gastrointestinal symptoms is explained by the longer presence of the virus in the gut. In addition, in immunocompromised patients’ diarrhea has been observed more frequently than in the general population.^23^^,^^24^ Therefore the persistence of diarrhea may be a marker of patients' altered immune function.

Because of this unexpected association between diarrhea and hospital readmission, the authors decided to evaluate factors associated with having diarrhea, trying to profile patients at high risk for complications. The authors only found younger age, diabetes mellitus, and female gender. The association between diabetes and the risk of infection and dysbiosis has been extensively described in the literature. The authors could not explain why younger age was associated with a higher risk for diarrhea.

One of the most intriguing findings in the present study was that the most frequent cause of hospital readmission was an infection. In a study from a Michigan database,^14^ 20% of patients who survived COVID-19 hospitalization were readmitted within 60 days after discharge. The most common readmission diagnoses were COVID-19 (30.2%), sepsis (8.5%), and pneumonia (3.1%). Because of the short period of time between discharge and readmission, the authors believe that COVID-19 reinfection is unlikely, thus it probably refers to post-COVID symptoms. The high occurrence of infection (sepsis and pneumonia) is similar to the findings in the present cohort. The authors believe that this topic deserves further investigation, such as the possibility of post-COVID-19 immunosuppression.

The present study has limitations. Most of the diagnoses were self-reported which may have led to inaccuracies. Second, it was not possible to confirm that these complications were a consequence of COVID-19 as there is no comparison group. Third, the authors do not have information regarding the time to hospital readmission. Finally, only 43% of the discharged patients returned for follow-up which might have led to bias.

In summary, the present data show that, for patients who required hospitalization for COVID-19, the presence of symptoms after hospital discharge was frequent, and there was a relatively high rate of readmission to the hospital. Significant factors for hospital readmission were the presence of previous co-morbidities, mechanical ventilation during COVID-19 hospitalization, and the presence of diarrhea. One of the most intriguing findings was that the most frequent cause of hospital readmission was an infection.

## Authors’ contributions

MPF, wrote the manuscript, data analysis; MSO, write the manuscript, data analysis; MMCM, write the manuscript; BMT, write the manuscript; IM, data compilation, data analysis; ACDSSN, write the manuscript; GFB, critical revision, study design; ASL, write the manuscript, critical revision, study design; HCFMUSP COVID-19 Study Group, data collection.

## Funding

This research did not receive any specific grant from funding agencies in the public, commercial, or not-for-profit sectors.

## Declarations sections

Ethical Approval: Protocols were approved by the local ethics committee (numbers 4.270.242, 4.502.334, 4.524.031, 4.302.745, and 4.391.560). Informed consent was obtained from all participants or their proxy prior to the study procedures and data publication. The personal information of participants is confidential.

Data availability statement: The data underlying this article will be shared on reasonable request to the corresponding author.

## Declaration of Competing Interest

The authors declare no conflicts of interest.

## Appendix Supplementary materials

Image, application 1
