# Supplementary material for: Frequency and factors associated with hospital readmission after COVID-19 hospitalization: the importance of post-COVID diarrhea
Source: Clinics (Sao Paulo). 2022 Jun 13;77:100061. doi: 10.1016/j.clinsp.2022.100061 (PMC9189119; doi:10.1016/j.clinsp.2022.100061)
Supplement: Supplementary file 1 [file mmc1.docx]

**CLINICS-D-22-00050_Supplementary Material**

**Supplementary Table 1** Evaluation of risk factors for diarrhea after discharge from COVID-19 hospitalization.

| **Variables** | **Diarrhea during follow-up** | | **RR (95% CI)** | **p-value** | **Multivariate analysis** | |
| --- | --- | --- | --- | --- | --- | --- |
|  | **Yes (n=184)** | **No (n=638)** |  |  | **OR (95% CI)** | **p-value** |
| Age in years Mean (range) | 54 (20‒101) | 57 (18‒92) | ‒ | 0.03 | 0.98 (0.97‒0.99) | 0.02 |
| Male sex | 83 (45%) | 354 (55%) | 0.91 (0.85‒0.98) | 0.01 | 0.68 (0.49‒0.95) | 0.02 |
| **Educational level** |  |  |  | 0.81 |  |  |
| 1 | 65 (35%) | 225 (35%) | ‒ |  |  |  |
| 2 | 30 (16%) | 118 (18%) | ‒ |  |  |  |
| 3 | 54 (29%) | 203 (32%) | ‒ |  |  |  |
| 4 | 26 (14%) | 78 (12%) | ‒ |  |  |  |
| BMI on hospital admission | 31 (20‒63) | 30 (14‒67) | ‒ | 0.06 |  |  |
| Smoking | 63 (34%) | 250 (39%) | 0.95 (0.89‒1.03) | 0.22 |  |  |
| **Variables associated with COVID-19 hospitalization** | | | | | | |
| Days of hospital stay median (range) | 15 (1‒154) | 12 (1‒123) | ‒ | 0.23 |  |  |
| WHO | 2 (1‒4) | 2 (1‒4) |  | 0.78 |  |  |
| Admission to ICU | 123 (67%) | 393 (62%) | 1.05 (0.98‒1.13) | 0.19 |  |  |
| Days of ICU stay median (range) | 5 (0‒126) | 3 (0‒100) | ‒ | 0.27 |  |  |
| Orotracheal intubation | 75 (41%) | 264 (41%) | 0.99 (0.92‒1.07) | 0.88 |  |  |
| Dialysis | 24 (13%) | 80 (13%) | 1.01 (0.90‒1.13) | 0.86 |  |  |
| **Underlying diseases** | | | | | | |
| Systemic arterial hypertension | 102 (27%) | 369 (4%) | 0.98 (0.91‒1.05) | 0.56 |  |  |
| Charlson score | 3 (1‒10) | 3 (1‒10) | ‒ | 0.38 |  |  |
| Chronic obstructive pulmonary disease | 11 (6%) | 33 (5%) | 1.04 (0.87‒1.24) | 0.67 |  |  |
| Asthma | 10 (5%) | 20 (3%) | 1.17 (0.91‒1.51) | 0.14 |  |  |
| Chronic kidney disease | 25 (14%) | 81 (13%) | 1.02 (0.91‒1.14) | 0.75 |  |  |
| Rheumatic disease | 52 (28%) | 175 (27%) | 1.01 (0.93‒1.10) | 0.82 |  |  |
| Sleep apnea | 30 (16%) | 107 (17%) | 0.99 (0.90‒1.09) | 0.88 |  |  |
| Hematologic disease | 5 (3%) | 11 (2%) | 1.12 (0.80‒1.56) | 0.55 |  |  |
| Diabetes mellitus | 82 (45%) | 214 (34%) | 1.12 (1.03‒1.21) | 0.006 | 1.77 (1.25‒2.51) | 0.001 |
| Cancer | 8 (4%) | 33 (5%) | 0.96 (0.82‒1.12) | 0.65 |  |  |
| Coronary heart disease | 20 (11%) | 81 (13%) | 0.96 (0.87‒1.07) | 0.51 |  |  |
| Congestive heart failure | 20 (11%) | 83 (13%) | 0.96 (0.86‒1.06) | 0.44 |  |  |
| Peripheral artery disease | 18 (10%) | 45 (7%) | 1.09 (0.93‒1.28) | 0.22 |  |  |
| Acute arterial occlusion | 3 (2%) | 6 (1%) | 1.17 (0.73‒1.85) | 0.43 |  |  |
| Thromboembolic events during hospital stay | 11 (6%) | 41 (6%) | 0.98 (0.85‒1.14) | 0.83 |  |  |
| Chronic liver disease | 9 (5%) | 38 (6%) | 0.96 (0.83‒1.11) | 0.58 |  |  |
| Sedentary lifestyle | 79 (43%) | 241 (38%) | 1.05 (0.97‒1.13) | 0.21 |  |  |
| **Social economic class** |  |  |  | 0.09 |  |  |
| A | 4 (2%) | 16 (3%) |  |  |  |  |
| B1 | 10 (5%) | 33 (5%) |  |  |  |  |
| B2 | 30 (16%) | 114 (18%) |  |  |  |  |
| C1 | 62 (34%) | 205 (32%) |  |  |  |  |
| C2 | 42 (23%) | 199 (19%) |  |  |  |  |
| D | 27 (15%) | 54 (8%) |  |  |  |  |

**Supplementary Figure 1** Distribution of Charlson score at hospital admission by decade of age of 822 COVID-19 patients.


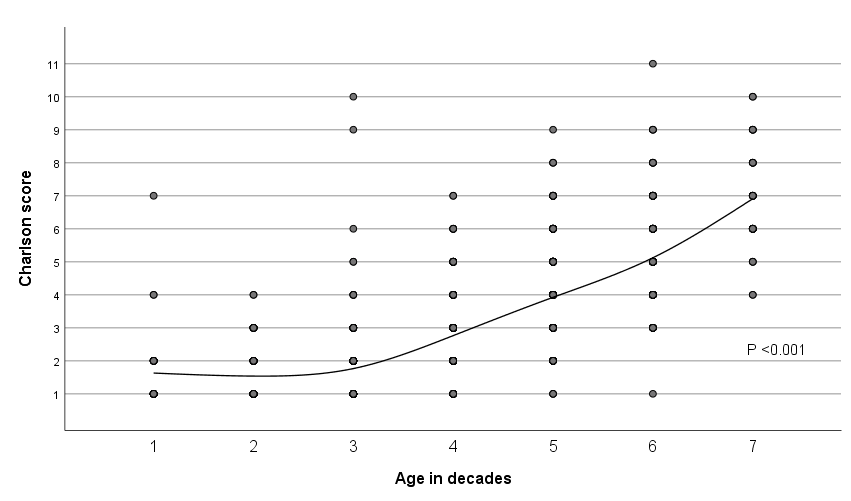


**Supplementary Figure 2** Distribution of self-reported infections occurring 6 o 11 months after discharge due to hospitalization for COVID-19.
